# A 'snip' in time: what is the best age to circumcise?

**DOI:** 10.1186/1471-2431-12-20

**Published:** 2012-02-28

**Authors:** Brian J Morris, Jake H Waskett, Joya Banerjee, Richard G Wamai, Aaron AR Tobian, Ronald H Gray, Stefan A Bailis, Robert C Bailey, Jeffrey D Klausner, Robin J Willcourt, Daniel T Halperin, Thomas E Wiswell, Adrian Mindel

**Affiliations:** 1 School of Medical Sciences, University of Sydney, Sydney, NSW 2006, Australia; 2 Circumcision Independent Reference and Commentary Service, 157 Stand Lane, Radcliffe, Manchester M26 1JR, UK; 3 Global Youth Coalition on HIV/AIDS, Pretoria, South Africa; 4 Department of African-American Studies, Northeastern University, Boston, MA 02115, USA; 5 Department of Pathology, School of Medicine, Johns Hopkins University, Baltimore, MD 21287, USA; 6 Research & Education Association on Circumcision Health Effects, Bloomington, MN 55425, USA; 7 Division of Epidemiology and Biostatistics, University of Illinois at Chicago, Chicago, IL, USA; 8 Divisions of AIDS & Infectious Diseases, University of California, San Francisco, CA 94122, USA; 9 Pregnancy Advisory Centre, The Queen Elizabeth Hospital, Adelaide, SA 5011, Australia; 10 Department of Education and Behavior, University of North Carolina School of Public Health, Chapel Hill, NC 27599, USA; 11 Center for Neonatal Care, Orlando, FL 32804, USA; 12 Sexually Transmitted Infections Research Centre, Westmead Hospital and University of Sydney, Sydney, NSW 2145, Australia

**Keywords:** Circumcision, Public health, Surgery, Infant health, Adolescent health, Foreskin, Urinary tract infections, Sexually transmitted infections, Penile cancer, Cervical cancer, Dermatology, Psychology

## Abstract

**Background:**

Circumcision is a common procedure, but regional and societal attitudes differ on whether there is a need for a male to be circumcised and, if so, at what age. This is an important issue for many parents, but also pediatricians, other doctors, policy makers, public health authorities, medical bodies, and males themselves.

**Discussion:**

We show here that infancy is an optimal time for clinical circumcision because an infant's low mobility facilitates the use of local anesthesia, sutures are not required, healing is quick, cosmetic outcome is usually excellent, costs are minimal, and complications are uncommon. The benefits of infant circumcision include prevention of urinary tract infections (a cause of renal scarring), reduction in risk of inflammatory foreskin conditions such as balanoposthitis, foreskin injuries, phimosis and paraphimosis. When the boy later becomes sexually active he has substantial protection against risk of HIV and other viral sexually transmitted infections such as genital herpes and oncogenic human papillomavirus, as well as penile cancer. The risk of cervical cancer in his female partner(s) is also reduced. Circumcision in adolescence or adulthood may evoke a fear of pain, penile damage or reduced sexual pleasure, even though unfounded. Time off work or school will be needed, cost is much greater, as are risks of complications, healing is slower, and stitches or tissue glue must be used.

**Summary:**

Infant circumcision is safe, simple, convenient and cost-effective. The available evidence strongly supports infancy as the optimal time for circumcision.

## Background

The English proverb "A stitch in time saves nine" teaches that to avoid a bigger problem later immediate effort is preferable to procrastination. Thus fixing a small hole in a sock with one stitch will avoid the need for nine stitches later when the hole becomes bigger. In the present article we consider whether this applies to medical male circumcision (MC) - referred to colloquially as a "snip".

Worldwide 1 in 3 males are circumcised [1,2], totaling an estimated 1.2 billion [2]. In the USA, medical MC is performed on 1.2 million newborns (56% of baby boys) in community hospitals annually [3,4]. The true number is higher because some boys are circumcised in ambulatory facilities, a physician's clinic or in a private home. In other developed countries infancy is also the most common time for performing MC, whereas in non-Muslim developing countries MC is usually part of coming-of-age ceremonies where risks are usually greater [5]. The largest number of circumcised males are Muslims (approx. 70% of circumcised males globally) [1].

Circumcision predates human history, with evidence of MC from art forms of the Upper Paleolithic period in Europe (38,000 to 11,000 years BCE) [6]. Rather than arising independently in diverse cultures globally [7], the practice more logically arose prior to the migration of *Homo sapiens *out of Africa [8]. If it had no survival advantage, it is unlikely that it would have persisted, and, as hypothesized by Cox & Morris, subsequent cessation of MC in some populations was perhaps a result of behavioral changes caused by environmental stressors or new religious philosophies such as Hinduism and Buddhism [8]. Such factors could explain why circumcision is relatively low in European, South and Central America, southern Africa, and non-Muslim Asian countries.

The awareness during Victorian times of a wide array of medical benefits from MC, including prevention of syphilis and better hygiene, led to a rise in its popularity in Anglo-Saxon populations in the 19^th ^century [7,9], continuing today in the USA in particular, where the majority of infant boys are circumcised [3,4]. In the UK circumcision is more common in the wealthier upper-classes, marking the fact that a doctor attended the birth rather than a mid-wife.

The advent of the AIDS epidemic in the 1980s re-focused interest on MC as a means of prevention of not just HIV, but other sexually transmitted infections (STIs) and adverse medical conditions. This has led to MC programs in high-HIV prevalence settings of sub-Saharan Africa focused on men for more immediate reductions in HIV incidence, but considerable interest has also been given to encouraging infant MC for longer-term gains [10,11]. There have as well been recent calls for the promotion of infant MC in the USA [12,13], the UK [14], Australia [15] and sub-Saharan Africa [16,17].

Despite the advantages of MC, few studies have directly compared the relative merits of MC at different ages. Here we present our findings after reviewing the literature, and document the relative pros and cons of infant MC versus MC in later childhood, adolescence or adulthood ("later circumcision"). We compare medical and surgical issues for infant versus later MC, attitudes and barriers, ethical issues, as well as cost-effectiveness. Our analysis has relevance to all countries, both developed and developing. Nevertheless, it should be recognized that a decision about circumcision is subject to varying considerations depending on the particular social and cultural context involved.

## Discussion

### Is infancy the best time medically?

Although an abundance of evidence exists about the benefits of MC [9,12,13,18], it is reasonable to ask whether these dictate infant MC rather than MC later in life when a boy can make up his own mind [19,20]. Some of the advantages of MC in infancy were featured in a report arising from an expert consultation conducted by the US Centers for Disease Control and Prevention (CDC) in 2007 [13]. Here we discuss several compelling reasons for infancy being the optimum time for MC.

An immediate medical benefit is the greatly reduced risk of a urinary tract infection (UTI), which is higher in infancy than any other year of life, and 10 times greater if the infant male is uncircumcised [21-26]. UTIs are common in uncircumcised infant boys [22-26] and cause severe pain. UTI as a cause of a fever at this age is often undiagnosed [27,28]. Bacteriuria in febrile boys presenting at hospital emergency departments occurs in 36% of uncircumcised boys, pointing to a UTI as the likely cause of fever, compared with only 1.6% of boys who are circumcised [29]. Antibiotic resistance in pathogenic bacteria under the foreskin is a growing problem [30]. The younger the infant, the more likely and severe the UTI will be, and the greater the risk of sepsis and death [31]. In the still-growing pediatric kidney [26,32] a UTI can result in permanent kidney damage in 34-86% of cases [33,34], thus exposing the boy to serious, life-threatening conditions later in life [26], including end-stage renal disease in 10% of cases [35]. In men, risk of UTI is over 5-fold higher if they are uncircumcised [36]. Thus infant MC offers protection against UTI over the lifetime.

**Figure 1:**
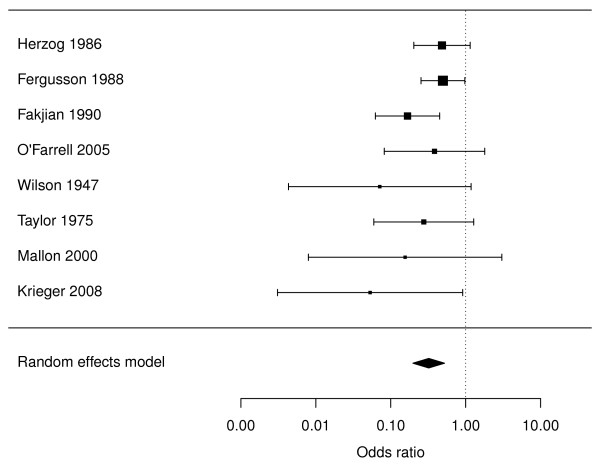
**Forest plot showing association between circumcision and penile inflammation in 8 studies **[38-45]. The meta-analysis shown does not include an anomalous outlier study [46], which when included led to significant between-study heterogeneity (*P *= 0.03), but when excluded no significant heterogeneity remained (*P *= 0.40).

Infant MC also offers immediate protection against inflammatory penile skin conditions such as balanitis, posthitis and balanoposthitis that are usually caused by *Candida *spp. [37]. Balanitis affected 5.9% of uncircumcised boys in one study [38] and 14% in another [39]. In male dermatology patients, balanitis was present in 13% of those who were uncircumcised compared to 2.3% of the circumcised [40]. After reviewing relevant studies [38-46] we conducted a meta-analysis to determine the level of protection against balanitis. This yielded an OR of 0.32 (95% CI 0.20-0.52) (Figure 1). Balanoposthitis was a cause of 26% of cases of acquired phimosis [47], in which the foreskin orifice is so narrow that the foreskin cannot be retracted. Lichen sclerosis, a chronic inflammatory dermatosis that results in white plaques and epidermal atrophy, is a disease of the uncircumcised male. It occurs in 35% [48] to 55% [49] of uncircumcised men with type 2 diabetes and peaks in the 30s [50]. Although most effectively cured by MC [50], it would be preferable to prevent it by MC in infancy. Delaying circumcision therefore results in greater exposure of the male to risk of penile inflammation.

All boys are born with phimosis. This resolves by about age 3 in all but approximately 10% of males, who as a result experience problems with micturition, ballooning of the foreskin, and painful difficulties with erections (see review [9]). Paraphimosis can similarly be prevented by infant MC.

Circumcision in infancy also means that by the time the male becomes sexually active, he has partial protection against those STIs known to be more prevalent in uncircumcised men [9,12,18,51,52]. Meta-analyses of observational studies show MC protects against oncogenic human papillomavirus (HPV) [53,54], genital herpes (HSV-2) [51], syphilis [51] and HIV [55]. The protective effect demonstrated by meta-analyses of the observational data [51,55] has, with the curious exception of syphilis, been reinforced by randomized controlled trials (RCTs) [55-61]). The trials also demonstrated increased efficacy to prevent HIV infection the longer the follow-up period after surgery. The protective effect is greater when MC is performed prior to sexual debut [51]. In men who have sex with men (MSM), while MC offers little protection against STIs acquired from receptive anal intercourse, MC does appear to protect men who are insertive-only, and to a similar degree as for vaginal heterosexual intercourse [62-64].

If the male is circumcised, his reduced vulnerability to carriage of several STIs means his female partner is less likely to become infected. The female partners of circumcised men are at reduced risk of HPV infection, the main cause of cervical cancer [53,65-67], as well as *Trichomonas vaginalis *[68] and bacterial vaginosis [68,69]. While RCT data were not as clear, observational studies have indicated that MC reduces female HSV-2 [70], *Chlamydia trachomatis *[71], and HIV [72-74].

MC timing has the same implications for all STIs prevented by MC. If a male becomes sexually active before he is circumcised, he is exposed to a period of increased risk of infection from several STIs. The length of this period varies according to the age at which circumcision is eventually performed. In countries with a high prevalence of STIs, the risk of infection before a male undergoes adult MC may be considerable. HPV and HSV-2 are an epidemic in virtually all countries worldwide [75,76]. Importantly, if a male has been circumcised in infancy or childhood, preceding sexual debut, the issue of infection with an STI during the post-MC healing period does not arise.

The risk of penile cancer is very much higher if a man is uncircumcised [54,77]. Many of the conditions above predispose to penile cancer. For example, meta-analyses found phimosis increases risk of penile cancer 12-fold (8 studies), balanitis 3.8-fold (4 studies) and smegma 3.0-fold (4 studies) [54]. These conditions are more common in or restricted to uncircumcised men. At least half of all penile cancers contain high-risk HPV types [78,79] and these can be an important predisposing factor [54]. A meta-analysis [53,54] and data from RCTs [60,80-85] have shown that MC protects against HPV infection. A very conservative meta-analysis noted that there were two-thirds fewer penile cancer cases in men circumcised in childhood [77]. It found the protective effect of MC may be greater for invasive than *in situ *penile cancer [77]. Because of lead-time bias and earlier diagnosis in a circumcised man, it was stated that the analysis was likely to have under-estimated the true protective effect of circumcision [77]. An association found between adult MC and penile cancer could be due to the fact that MC when performed in adulthood is frequently to remove cancerous lesions or to treat conditions such as phimosis and recurring balanoposthitis that themselves are associated with predisposition to penile cancer. Therefore the association does not necessarily imply that delaying MC to adulthood increases the risk of penile cancer.

There is also some evidence that MC protects against prostate cancer, a malignancy associated with a history of STIs (see reviews [9,54,86]).

Arguments that benefits and risks of MC are evenly matched are not supported by an analysis of the frequency of each, as shown in Table 1, which also indicates grade of quality of the evidence [87]. Even though MC in adults still provides many benefits, and is currently a crucial intervention in the high-HIV-prevalence epidemics of sub-Saharan Africa, where many men are at considerable risk of acquiring HIV, when considering all of the conditions MC protects against, the benefits of performing this procedure in infancy predominate over later circumcision (Table 2). When aggregating the frequency of each condition that is higher in uncircumcised males, it has been calculated that as many as half of uncircumcised males will, over their lifetime, require medical attention for at least one of these conditions (Table 1). Thus immediate, as well as assured lifetime protection against a range of adverse medical conditions and infections supports infancy as the optimum time to perform circumcision.

**Table 1:** A comprehensive risk-benefit analysis of infant MC

Risks from not circumcising			
*Condition*	Level of evidence*	*Fold increase*	*NNT*^†^
Urinary tract infection (infants)	1++	10	50
Urinary tract infections (lifetime)	2+	5	4
Pyelonephritis (infants)	2+	10	100
- with concurrent bacteraemia	2+	200	1000
- childhood hypertension	2	-	1500
- end-stage renal disease (lifetime)	2+	-	500
Candidiasis	2	2	10
Prostate cancer	2	1.5-2	6
Balanitis	2++	3	10
Phimosis	1++	infinite	10
High-risk HPV	1++	3	2
Genital herpes (HSV-2)	1+	1.35	
Syphilis	1+	3	200
HIV infection	1++	3-8	1000
Penile cancer	1++	> 20	1000
***In female partner:***			
Cervical cancer	1++	4	-
Chlamydia	2+	4	-
HSV-2	2+	2	-
Bacterial vaginosis	1+	2	-
Thus risk in an uncircumcised male of developing a condition requiring medical attention over their lifetime = 1 in 2			
**Risk associated with medical MC in infancy**			
***Condition***	***Fold increase***	***NNH***^††^	
Local bruising at site of injection of local anesthetic (if dorsal penile nerve block used)	0.25*	4	
Infection, local	0.002	600	
Infection, systemic	0.0002	4000	
Excessive bleeding	0.001	1000	
Need for repeat surgery (if skin bridges or too little prepuce removed)	0.001	1000	
Loss of penis	close to 0	1 million	
Death	close to 0	Over 1 million	
Loss of penile sensitivity	Low	High	
Thus risk of an easily-treatable condition = 1 in 500 and of a true complication = 1 in 5000			

*As per Scottish Intercollegiate Guidelines Network (SIGN) grading system for evidence-based guidelines [87], which ranges from 1++ (highest) to 4 (lowest).

Values shown are based on statistics for USA (for source data see review [18] and references cited in the present article)

Abbreviations:

^†^*NNT *number needed to treat - i.e., approximate number of males who need to be circumcised to prevent one case of each condition associated with lack of circumcision.

^††^*NNH *number needed to harm, i.e., approximate number of males that need to be circumcised to see one of each particular (mostly minor) adverse effect. *The minor bruising (from this method only) disappears naturally without any need for medical intervention, so is not included in overall calculation of easily-treatable risks

**Table 2:** Approximate figures for benefits of circumcision in infancy versus circumcision later

***Condition***	***Infancy***	***Later***	***Critical age for maximum benefit***
UTI	10 ×	5 ×	birth, highest risk in 1^st ^year of life
Phimosis	5-infinity	5-infinity	birth
Balanitis	3 ×	3 ×	birth, higher risk after onset of sexual activity
Hygiene	n/a	n/a	birth
HIV	3-8 ×	3-8 ×	onset of sexual activity
HPV	2 ×	2 ×	onset of sexual activity
HSV-2	1.3 ×	1.3 ×	onset of sexual activity
Thrush	2 ×	2 ×	onset of sexual activity
Penile cancer	3-22 ×	less	protection level unclear if performed after childhood

See main text for references to each condition

While the medical evidence supports infancy as being the optimum time to circumcise, it is recognized that instituting infant circumcision might present a challenge to individuals in cultures in which circumcision is an important part of coming-of-age ceremonies or that are traditionally opposed to circumcision, particularly in countries in which circumcision is a mark of religious affiliation (e.g., Hindu versus Muslim).

### Is infancy the best time surgically?

Evidence clearly shows that circumcision in infancy carries fewer risks of complications than circumcisions performed in childhood or later in life. In infancy, surgical complications for large published series range from 0.2% to 0.6% [23,88-90]. Higher rates of 2-10% have been reported in much older and smaller studies [91-93]. A recent systematic review found a median complication frequency of 1.5% among studies of neonatal or infant circumcision, compared to 6% among studies of children aged one year or older [94]. Almost all of such complications are minor and can be easily - and completely - treated. In both infants and older children, severe complications (as compared to mild complications) were rare, with a median frequency close to zero [94].

While excluded from systematic review, the frequency of complications among adult MC patients was noted to be higher than the frequency of complications from MC in children older than 1 year [94]. In the large RCTs of adult MC, complications were seen in 1.7-3.8%; these were virtually all mild or moderate and were effectively treated [56-58] (Table 3).

**Table 3:** Complications and their frequency for medical MC of men in RCTs in South Africa (3.8%, all mild or moderate), Kenya (1.7%, all mild or moderate) and Uganda (4% mild, 3% moderate [breakdown not disclosed] and 1% severe [shown])

Condition	South Africa	Kenya	Uganda
Bleeding post-op	0.6%	0.4%	0.08%
Infection	0.2%	0.4%	0.04%
Wound disruption	0%	0.3%	0.04%
Delayed healing	0.1%	0.2%	-†
Swelling or hematoma	0.6%	0.1%	-
Severe pain	0.8%	0%*	-
Appearance problem	0.6%	0%	-
Damage to the penis	0.3%	0%	-
Too much skin removed	0%	0%	-
Too little skin removed	0.3%	0%	-
Anesthesia-related event	0.06%	0.1%	-
Problem urinating	0%	0%	-
Other	0.3%	0.4%	-
Death	0%	0%	-

Refs: [56-58], respectively

*At the 3-day post-operative visit pain was zero in 48% of men, mild in 52% and severe in none

†Dashes indicate that the item was not reported in the publication

Another issue is a fear of complications - whether real or imagined - when circumcision is performed later. Such fears can be a significant barrier to uptake of adult MC. In a US study, 59% of men expressed worries about risks of bleeding and infections [95]. A study in China found that 12.5% of men were concerned about infection [96]. Education about the actual low frequency of complications is thus necessary to allay such fears.

Other desirable features of infant MC are the surgical ease of performing a circumcision on an immobile newborn, the speed of the operation, absence of any need to use sutures, quick healing, and good cosmetic outcome [97,98]. Further information is provided in an extensive recent review of instrumentation and techniques for infant and later circumcision [99].

When the frequency and severity of complications from the procedure itself are compared with the frequency and severity of medical conditions, including deaths, that can result from not circumcising, the evidence strongly favors the argument for MC in infancy [9] (Table 1). Nevertheless, circumcision later is far better than no circumcision at all.

### Parental acceptability of MC in infancy

Despite infancy having a favorable risk-benefit ratio for MC, parents must make the ultimate decision over whether to circumcise infant sons or not. A survey in the USA found that 88% of participants were willing to circumcise a son [100]. A review of 13 studies in 9 sub-Saharan African countries found a median of 81% (range 70-90%) of women would choose to circumcise their sons [101]. After an informational session about MC, 74% of men in the Dominican Republic expressed a willingness to have their sons circumcised [102]. In India, a study of women, 78% of whom were Hindu (a religious group that does not traditionally circumcise), found that after being informed about risks and benefits, 81% said they would definitely have their boy(s) circumcised if the procedure were offered in a safe hospital setting, free of charge [103]. Only 1% said they would definitely not have their boy circumcised [103]. In general, when choosing when it should be carried out, the neonatal period or childhood appears to be more acceptable than MC later.

Unfortunately, in a survey in California, 40% of parents believed they had not been provided with enough information about MC to make an informed choice [104]. For parents of boys who were not circumcised, the doctor had not discussed circumcision with them, as opposed to 15% of parents of boys who were circumcised. Twice as many parents would, in retrospect, have wanted their boy to have been circumcised had they known more. After reading information about MC, 86% of parents were in favor of neonatal circumcision [105]. Overall, support was higher among parents born in the USA, but lower among Hispanic parents.

The reasons for MC given by Australian parents include family tradition, improved hygiene and reduced risk of diseases and other conditions that MC protects against [106]. A study of African-American parents found that 96% strongly believed pediatric circumcision to be healthy, and 73% considered it essential [107]. Interestingly, the study found that it was the mothers who most often made the final decision. This demonstrates the need to engage and educate mothers and pregnant women about MC for their infant boys.

### Acceptability of adult MC

MC does have benefits at later ages, but a man must be willing to avail himself of these by getting circumcised. It is therefore important to examine the acceptability of MC by adult males. In the USA, only 13% of uncircumcised heterosexual men indicated that they would be willing to become circumcised to lower their risk of HIV [108]. In sub-Saharan Africa, however, where HIV is an epidemic, an extensive review of 13 studies found that a median of 65% (range 29-87%) of heterosexual men were willing to be circumcised [101]. Men and women in a Kenyan study exhibited a good understanding of the need to maintain safe sexual practices [109]. In India, of 467 uncircumcised heterosexual men in a high-HIV prevalence region, 93% agreed that men should consider MC for HIV prevention, and 58% would accept free medical MC [110]. Facilitators of acceptability included improved penile hygiene (97%), reduced HIV/STIs (91%), lower risk of penile cancer (90%) and of cervical cancer in their female partner (86%) [110]. In Kenya, perceived improvement in sexual pleasure was a facilitator [109,111]. In the Dominican Republic willingness was only 29% initially, but after an information session explaining the risks and benefits of the procedure, this figure increased to 67% [102]. Acceptability in Thailand was 14%, rising to 25% after an information session [112]. In a Chinese study, 39% were willing to be circumcised to protect themselves from infection, and 46% would consider it to protect their partner as well [113]. In other samples of mostly heterosexual Chinese men, 41% were willing to be circumcised in one study [114] and 25% in another [115].

In studies of MSM, a US study found that 53% of participants were willing to be circumcised in one survey [95], whereas another, conducted in San Francisco, found 28% of the uncircumcised were willing to get circumcised if there was evidence of efficacy, but only 0.9% of those for whom MC would be a relevant intervention (mostly those who engaged in insertive anal intercourse not using condoms) were willing [116]. In Scotland, only 14% of MSM indicated their willingness to take part in a circumcision trial [117]. One study in China found 43% of MSM were willing to be circumcised [96], and in another, 8% were willing initially, but this rose to 31% after an information session [118]. The lower rates of acceptability among MSM compared to heterosexual men could be due to the fact that recent studies of MC have not shown a benefit for most MSM in protection against HIV [63,119]. However, these studies included men who were both receptive and insertive anal sex partners, and MC only offers protective benefits for MSM who are mostly or exclusively insertive [63,119].

Even if a man is willing to be circumcised this does not mean he will end up having the procedure done. On the other hand, a lack of willingness to be circumcised should not be interpreted as a preference to be uncircumcised. This is because a large number of obstacles have been documented, such as fear of pain or complications, embarrassment, inconvenience and cost. The obstacles are discussed in the following sections. It is reasonable to suppose that, if these barriers could be addressed through the provision of correct information and financial assistance, the fraction of men willing to be circumcised would increase significantly. Better education of parents before or soon after their baby is born about actual risks should, by helping to ensure a circumcision in infancy, avoid later deliberations and barriers to circumcision in adolescence and adulthood.

### Barriers

#### Pain

Since not all men are willing to be circumcised, even when their infection risk from not doing so may be high, there are clearly barriers to an affirmative decision, particularly in high HIV prevalence settings where MC is being rolled-out to reduce infections.

In a review of 13 acceptability studies of heterosexual men in sub-Saharan Africa, concern about possible pain was "the major barrier" to agreeing to be circumcised [101]. As well as pain, the long healing period, meaning no sex, and MC not being part of the local culture, were other impediments to getting a circumcision [109,111]. In Pune, India 71% of men expressed this concern [110]. Amongst MSM, fear of pain was a barrier for 62% of men in the USA [95] and was 47% for Chinese men [96]. An acceptability study among African-American parents found that despite high (88%) perception of pain in their child, 73% strongly believed that MC was necessary [107].

In practice, the pain associated with medical MC is far less than men anticipate, and many are not aware that local anesthesia is recommended. In the large RCTs, severe pain was reported in only 0.8% of 1,568 participants in the South African trial [56], 0.3% of 2,326 HIV-negative men and 0.2% of 420 HIV positive men in the Ugandan trial [120], and in the Kenyan trial, of 1,334 men, "very mild" pain was reported in 52% at postoperative day 3 and 11% at day 8, with none of the men reporting pain more severe than "very mild" [57]. In a small trial of the "Shang Ring" device used to circumcise 40 men, pain scores (graded from 0 = no pain to 10 = worst possible pain) averaged 3.5 during erections [121]. Since erections would place the most tension on the wound during healing, erections likely contribute maximally to pain scores.

It is instructive to consider here the issue of pain associated with an infant circumcision. In infancy, local anesthesia is effective in reducing or almost eliminating pain during and after circumcision [122], although gauging the level of pain experienced is more subjective than what can be ascertained from communications by older children or men. Of interest is that neonates exhibit lower pain scores than older infants [123]. Their response to pain in general is less when delivered vaginally than by cesarian section [124]. As an aside, early exposure to noxious or stressful stimuli decreases pain sensitivity and behavior in adult life [125,126]. While there may be some short-term memory of pain [127], no credible study has been conducted into long-term memory of pain experienced in infancy. Irrespective of such considerations we strongly support a recommendation of adequate pain control as being essential during and after a circumcision at any age.

Thus, although pain is overall minor and should not be seen as a major barrier, the fear of pain for later circumcision does represent a significant barrier.

#### Cost

Acceptability studies show cost to be a frequent barrier to adult MC [101], although willingness is higher if costs are borne by others. The barrier of cost, especially for poor families, has not been helped by an unscientific (but successful) lobbying campaign by MC opponents that led 18 states in the USA to eliminate coverage for circumcision by Medicaid, the public insurance program that insured 50.3 million people as of June 2010, or about one of every six Americans [128-130], and that led to a ban on elective MC in public hospitals in all but one state in Australia. While immediate costs to the health system might have been reduced, the longer-term costs for medical need and conditions caused by lack of circumcision can only be greater [131,132].

The cost of a neonatal circumcision is far lower than circumcision later [98]. Cost estimates in the USA for a circumcision are approximate $165 [131] to $257 [133] in infancy, compared with approx. US$1,800-2,000 for circumcision in adolescence or adulthood [131,134]. Even if the adolescent or adult male wants to be circumcised, the cost can be prohibitive. Cost can be reduced by insisting on a local anesthetic, since a general anesthesiologist's fees can be considerable. In developing countries, the cost of a circumcision is typically US$59 for adults or adolescents, and US$15 for newborns [11].

Although the costs are greater in developed nations, when represented as a fraction of GDP per capita [135], the figures are comparable between each: 0.4%-1.4% of GDP per capita for neonatal and 4.2%-5.4% for MC in adolescents or adults. Health interventions are considered highly cost-effective at a threshold below 1% of GDP per capita [136]. Thus the cost of adult MC represents a significant sum. Affordability of MC is not helped by the lower earnings typical of younger men. In developing countries, the extreme poverty of many people means any cost is unaffordable by most of the population.

While MC protects against numerous conditions and infections, in the case of HIV, in locations where HIV prevalence is high and MC rates are low, increasing adult MC should be regarded as an urgent objective, while increasing infant MC should be an important objective. In populations where HIV prevalence is still low and MC rates are low, increasing infant MC should be a priority.

### Cost-effectiveness

In a cost-benefit analysis in the USA it was found that, for a range of medical conditions, "much of the initial cost of neonatal circumcision is eventually recovered when disease and the medical need [in 9.6% of males] for post-neonatal circumcision are prevented" [131]. This analysis was criticized as being overly conservative [132].

In the case of HIV reduction, modeling in high-prevalence settings such as sub-Saharan Africa has shown that adult MC would be highly cost-effective [137,138]. Similarly, neonatal MC was calculated to provide enormous cost savings in populations where HIV prevalence is high [11]. Net cost per HIV infection averted in Rwanda was US$3,932 for adolescent circumcision and US$4,949 for adult circumcision [11]. Reviews of 21 [139] and 5 [140] cost-effectiveness studies found adult MC to be very cost-effective, the cost per HIV infection averted ranging from US$174 to US$2,808 [140]. MC was particularly cost-saving after due consideration of the cost of HIV treatment, treatment cost being estimated as US$2.3B over 20 years [141].

In low prevalence settings it has been argued that MC is a waste of money as it will have little impact on HIV [142-144]. This may not be true, however, as shown by CDC calculations that found infant MC to be cost-saving for future HIV prevention in Black and Hispanic males in the USA, although not in non-Hispanic White males, perhaps because the latter have the highest MC rates and much lower HIV prevalence [133].

### Cosmetic outcome

When circumcision is performed in infancy the ability of the inner and outer foreskin layers to adhere to each other means sutures are rarely needed and the scar that results is virtually invisible [98]. Other factors include the more rapid healing at this time of life, contributed by age-associated differences in pro-inflammatory factors that might affect scar formation [145].

In studies on adult MC, both men and their partners preferred the new appearance of the penis post-circumcision [146,147]. In the case of MSM, in a Chinese study, only 2.5% of men expressed concern about cosmetic outcome [96]. Despite the fact that MC rarely causes permanent disfigurement from scarring when performed properly, the fear of a poor cosmetic outcome is a documented deterrent of acceptability. For example, a study in the South American Andes found that MSM identified the risk of scarring as a significant barrier to MC [148].

### Sexual function and activity

The effect of an infant circumcision on sexual function and activity cannot be determined directly, but can be inferred from studies of men circumcised as adults. Numerous studies show that MC has no adverse effect on sexual function [147,149-152]. This finding is supported by data from the large RCTs in sub-Saharan Africa [45,153] which included more than 10,000 participants. A study in Turkey found no relationship between age of childhood circumcision and overall sexual function in men aged 22-44 [154]. Since all men are circumcised, mostly in childhood, in this Muslim country there was no control group of uncircumcised men to compare with. Of seven areas of sexual function examined (frequency of intercourse, communication, degree of satisfaction, avoidance, sensuality, ejaculatory function and erectile function), the only difference was lower avoidance in those circumcised between the ages of 0-2, compared to the 3-5 and 6-12 age groups [154]. A study of MSM in Sydney reported that later circumcision was associated with erectile dysfunction and premature ejaculation difficulties in some men [155]. Such difficulties were not seen in men who had been circumcised in infancy. In developed countries, most later circumcisions tend to be for treatment of a medical condition and this could offer a partial explanation for the finding. Since men circumcised later were less likely to engage in insertive anal intercourse, psychological effects after MC for medical need, at an age where the male has cognitive awareness of his previous painful penile problems, as well as the surgery itself, seemed a probable explanation. In a large Danish study in which circumcision, mostly for medical reasons, accounted for the small proportion of circumcised men surveyed, there were no differences in a range of sexual measures, apart from a statistically questionable [156] difference in ability to reach orgasm during intercourse in a minority of 10 circumcised men [157].

When circumcision is delayed beyond the onset of sexual activity, the impact of a period of abstinence must be considered. Analysis of data from three RCTs found that relatively few men engaged in sexual intercourse within 42 days of circumcision [158]. It has been suggested, not unreasonably, that this period of complete abstinence (from both intercourse and masturbation) is "often daunting and serves as a disincentive for men to undertake the procedure" [159], and the recommended post-surgical abstinence period was found to be a significant barrier to MC uptake in Kenya [111]. Circumcision in infancy, or indeed at any time before puberty, eliminates such an obstacle.

### Sexual pleasure

A range of beliefs exists about the effect of MC on sexual pleasure and function. A comprehensive review of acceptability studies in sub-Saharan Africa noted that men who were willing to be circumcised considered that MC would not adversely affect sexual pleasure [101]. Subsequent surveys support this, with many men considering that MC will enhance their sexual performance and satisfaction [111]. However, a belief that MC might reduce their sexual pleasure was the reason 46% of men in a Dominican Republic study were reluctant to be circumcised [102], as was also the case for 14% of men in an Indian study [110], and 5.3% of men in a Chinese survey [96]. In the latter study approximately three times as many men thought circumcision would increase, rather than diminish, their sexual pleasure [96]. In the USA, 18% of men said they would consider circumcision because it might increase sexual pleasure, this being associated with willingness to be circumcised [95]. In another US study, 35% of African American parents thought circumcision increases pleasure, although this was not a significant factor in deciding on circumcision for their boys [107].

Fears and anxieties about sexual pleasure appear to be substantial. This may be especially problematic in developed countries with widespread Internet access, as this medium is dominated by anti-circumcision websites, many of which spuriously claim that MC severely harms the sexual experience. This was documented in a survey of 73 Internet sites devoted to MC [160].

Scientific evidence regarding the sexual effects of MC does not substantiate the purported harms to sexual pleasure. The better-quality studies (in terms of sample size, rigor of methodology, accuracy of analysis of findings, and generalizability of results) have found no adverse effect of MC on penile sensitivity [151,161-163], sensation during arousal [164], sexual satisfaction [146,151], premature ejaculation [165], intravaginal ejaculatory latency time [166,167], or erectile function [147,149-152]. Two RCTs found MC does not adversely affect sexual function, sensitivity or satisfaction [45,153], with one of these studies showing that the sexual experience of most men was enhanced after circumcision [45]. Some studies have found that MC reduced the risk of premature ejaculation [168,169].

In several studies, perceptions about partners' sexual pleasure and preferences were also important predictors of willingness to be circumcised [101]. A study of Chinese MSM found that 15% thought MC would improve the partner's sexual pleasure, while 4% thought it would decrease it, and 68% were unsure [96]. In sub-Saharan Africa, 69% (range 47-79%) of women preferred circumcision for their partners because of its perceived aesthetic value [101], consistent with credible studies in developed countries [170,171].

Credible studies of the female partners of adult MC patients have found no adverse effect on sexual experience. For example, data from 455 women in a Ugandan RCT indicated no change (57%) or an improvement (40%) in sexual satisfaction after their male partner had been circumcised [172] and a Mexican study found no change in sexual satisfaction, desire, pain during vaginal penetration or orgasm [173]. A study in Sydney of MSM found no overall differences between the circumcised and uncircumcised in participation in insertive or receptive anal intercourse, difficulty in using condoms, or sexual problems such as loss of libido [155]. A survey of US women found 82% preferred the circumcised penis for fellatio, with only 2% preferring the uncircumcised penis [170].

The fact that circumcision does not impair - and for many may enhance - a man's sensation and sexual pleasure, should reassure men considering whether to get circumcised [174]. It should also reassure parents who may wonder about this issue when deciding to have their infant son circumcised.

### Psychological consequences

Very few credible studies have examined psychological factors associated with MC.

A study of Californian boys in their early teenage years found that circumcised boys - the majority of whom were circumcised neonatally - were more satisfied with their circumcision status than were uncircumcised boys [175]. A study in Sweden, where MC is uncommon, found no serious psychological disorders amongst boys circumcised in childhood, although shyness in the change-room was noted in 7% [176].

An acceptability study conducted in the Sichuan province of China found 53% of men were concerned that MC would be "too sensitive and embarrassing" [114]. Concerns were also expressed that men might be mocked for undertaking the surgery.

In India, where MC is a mark of religious affiliation, 41% of mostly Hindu men were concerned that MC was not part of their culture, while 30% were afraid of stigma or rejection [110]. MC has historic implications in India, where Muslim men were targeted for violence based on their circumcision status during the Hindu fundamentalist, anti-Muslim pogroms of 2002 and subsequent riots [177]. It has been suggested that MC in India might be more acceptable to STI clinic attendees than others [178].

Psychological effects were the probable explanation for findings in MSM that later circumcision, usually performed to treat a medical problem, was associated with lower insertive anal intercourse [155]. As referred to earlier, this is likely because, when older, the male has cognitive awareness of his previous painful penile problems, as well as the surgery itself.

There is some concern about risk-compensation (the tendency to stop using condoms and increase the number of sexual partners) following MC, although in most studies in which men were counseled this was not seen [179,180]. It has been suggested that neonatal MC may reduce the chances of a change in behavior due to circumcision status, as the male will not perceive any change in risk compared to what might transpire if the circumcision had taken place at an age when he might be sexually active [181].

While these various psychological problems should be mitigated by making MC normative in a community, just as with most fears and anxieties, the prospect of such concerns would be largely eliminated if MC were performed in infancy.

### Absence from work or school

Unlike the convenience of circumcising a baby that sleeps most of the time and is a dependent in society, circumcision during productive work or school years will typically require taking time off, although the amount of time off required is typically small. In one study of men circumcised with the Shang Ring device, men took an average of 1.1 days off work; 80% were back at work by day 2, with only 20% requiring more than 2 days, and little disruption to activities or discomfort was reported for the week the ring was in place [121]. Eighteen percent of men in the study reported disruption to their work while the device was present, and 30% had not resumed routine leisure activities by 7 days. In the large Kenyan RCT, only 4% of men required 3 days or more before they could return to normal activities [57]. In a study of childhood MC, median times of 5 days to return to normal activity and 7 to return to school have been reported [182]. This may have been because children are usually more active than adults, thus increasing the chances of injury and so prolonging the healing period.

### Ethical considerations

Nowhere is MC illegal. Concern has, however, been expressed by some authors about the ethical implications of circumcising boys who are too young to give consent [19,20]. The "autonomy-centered" argument of these authors is that MC should be delayed until the individual can decide for himself. But it has been pointed out that this argument is not consistent with the rationale behind other interventions, such as vaccinations, which are similarly performed before the child is old enough to consent and which carry similar risks of complications [183-185]. The authors of one bioethical analysis concluded that MC is appropriate for parental discretion [184]. Other bioethicists have argued that MC in the face of high risk of infection and disease is ethically imperative, as to do otherwise would risk human lives [17] and under such circumstances MC should be regarded as a justifiable public health measure [185]. Given the high infection and disease risk overall to the male and his female partners (Table 1) there would be few populations in the world that would not benefit from MC.

## Conclusions

Infancy presents a "window of opportunity" for circumcision. It is associated with substantially lower costs, lower risk of complications when performed by an experienced operator in a clinical or other appropriate setting, and lower lifetime risk of a variety of adverse conditions and infections [186]. The health benefits include protection against urinary tract infection and thus permanent damage to the still-growing kidney, reduced likelihood of penile inflammation, and elimination of risk of phimosis, which impedes micturition and results in difficult and painful erections in adolescence and adulthood. It also means tearing of the fragile foreskin and frenulum is avoided. Circumcision means an assurance of greatly reduced risk of penile cancer later in life, no smegma, better hygiene, and lower risk of various STIs. These not only include HIV that is an epidemic in some locations, but also oncogenic HPVs and genital herpes that are an epidemic worldwide. In the future female sexual partners of males, infant MC means they too will be at reduced risk of STIs and cervical cancer.

Some of the arguments against waiting until later to circumcise are:

• Protection against UTIs and damage to the fragile pediatric kidney is lost.

• Infant MC eliminates risk of phimosis and balanitis in childhood and after puberty.

• If circumcision is performed after boys become sexually active benefits associated with STI prevention are delayed.

• The risk of complications is higher for later circumcisions.

• The cost (to the individual or the public purse) is much higher, and often unaffordable, for later circumcision.

• Educational resources for boys to make an informed decision are quite limited.

• Large-scale adolescent circumcision would strain medical resources.

• Boys who later choose circumcision will likely wish it had been performed in infancy.

• Many older boys and men may not want to face an operation even though they wish to be circumcised.

• The momentum amongst major international and American health and medical organizations towards encouraging circumcision, especially in infancy.

Circumcision in infancy avoids any embarrassment of having it done later, as well as anxieties about pain, complications and adverse sexual effects, even though these are minimal or not supported by evidence. It also avoids arguments about whether there might be adverse psychological consequences for MC performed later in childhood. And absence from work or school is avoided.

There are fewer barriers to MC in infancy. The infant is less mobile, so facilitating the use of local anesthesia, the procedure is simpler, healing is quicker, the cosmetic outcome is superior, and cost-effectiveness is high, as is acceptability. The neonatal period should therefore be regarded as the optimal time to perform circumcision. It is viewed as a vital component of public health strategies aimed at realizing high levels of MC in the population [187]. The procedure should be performed by a trained professional using appropriate local anesthesia in a clean environment. Circumcision outside of such a setting is ill-advised, so explaining why clinical MC is increasingly being made available in European countries to Muslim families.

We recommend that evidence-based policies be developed regarding the availability of infant MC in all countries worldwide. It has been suggested that policies surrounding neonatal MC should be integrated into existing health systems as part of postnatal care [183], with adolescent and adult MC constituting "catch-up" campaigns that would be phased out over time [11]. This should not detract from the immediate urgent need for safe voluntary adult medical MC services in high-HIV-prevalence regions in particular.

## Competing interests

The authors declare that they have no competing interests.

## Authors' contributions

BJM and JHW drafted the manuscript. JHW performed the statistical analyses shown in Figure 1. BJM, JHW, JB, RGM, AART, RHG, SAB, RCB, JDK, RJW, DTH, TEW and AM made substantial contributions to successive drafts and thereby to the intellectual content of this article. All authors read and approved the final manuscript.

## Pre-publication history

The pre-publication history for this paper can be accessed here:

http://www.biomedcentral.com/1471-2431/12/20/prepub
